# Supplementary material for: Relevant, Hidden, and Frustrated Information in High-Dimensional Analyses of Complex Dynamical Systems with Internal Noise
Source: J Chem Theory Comput. 2025 Jul 2;21(14):6683–97. doi: 10.1021/acs.jctc.5c00374 (PMC12288070; doi:10.1021/acs.jctc.5c00374)
Supplement: Supplementary file 1 [file ct5c00374_si_001.pdf]

# Relevant, Hidden, and Frustrated Information in High-Dimensional Analyses of Complex Dynamical Systems with Internal Noise

Chiara Lionello[1], Matteo Becchi[1], Simone Martino[1], and Giovanni M. Pavan*[1]

[1]Department of Applied Science and Technology, Politecnico di Torino, Torino 10129, Italy

June 20, 2025

*Corresponding author: giovanni.pavan@polito.it

## Supporting Information

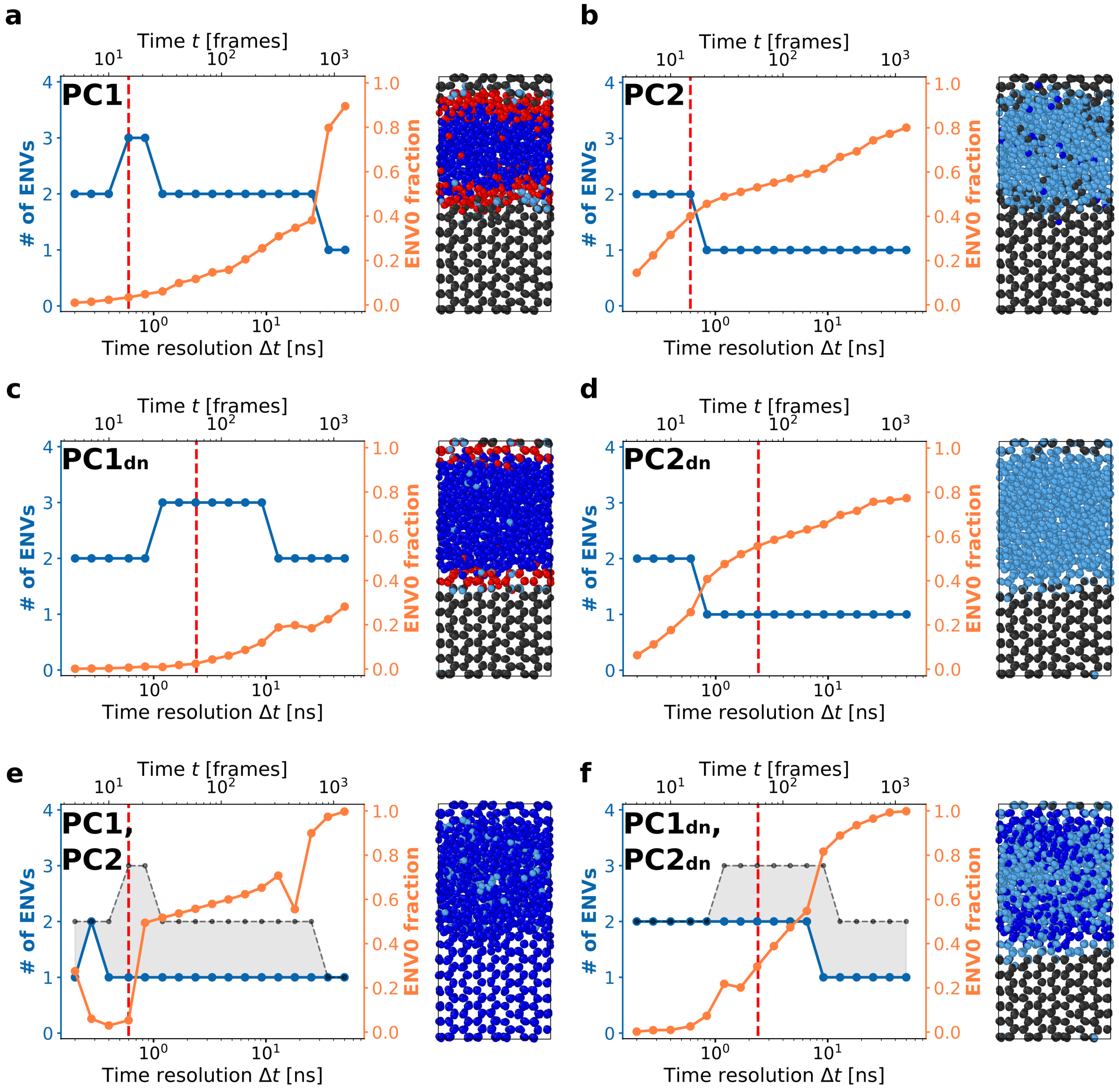


Figure S1: **Onion Clustering on PC1 and PC2 time-series data, obtained from SOAP with $l_{max} = n_{max} = 4$.** a) and b) Onion Clustering results calculated for raw PC1 and PC2, and relative screenshots. c) and d) Onion Clustering results on PC1 and PC2 denoised, and relative screenshots. e) and f) Results from the Onion Clustering algorithm applied on the bi-dimensional (PC1, PC2) time-series, e) raw and f) denoised.

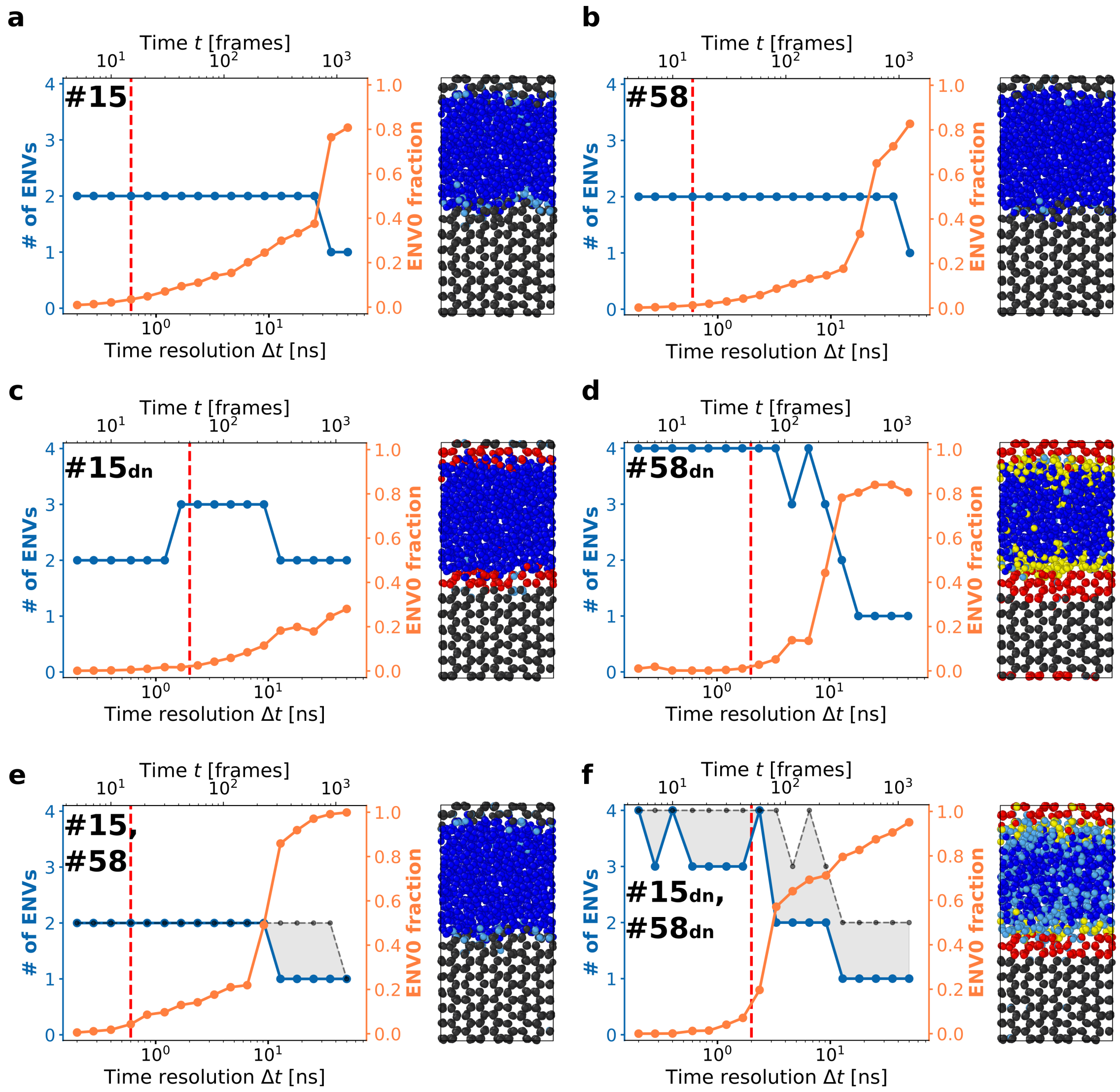


Figure S2: **Onion Clustering on components #15 (highest variance for $l = 0$) and #58 (highest variance for $l > 0$), both raw and denoised.** The figure follows the same structure of Figure S1.

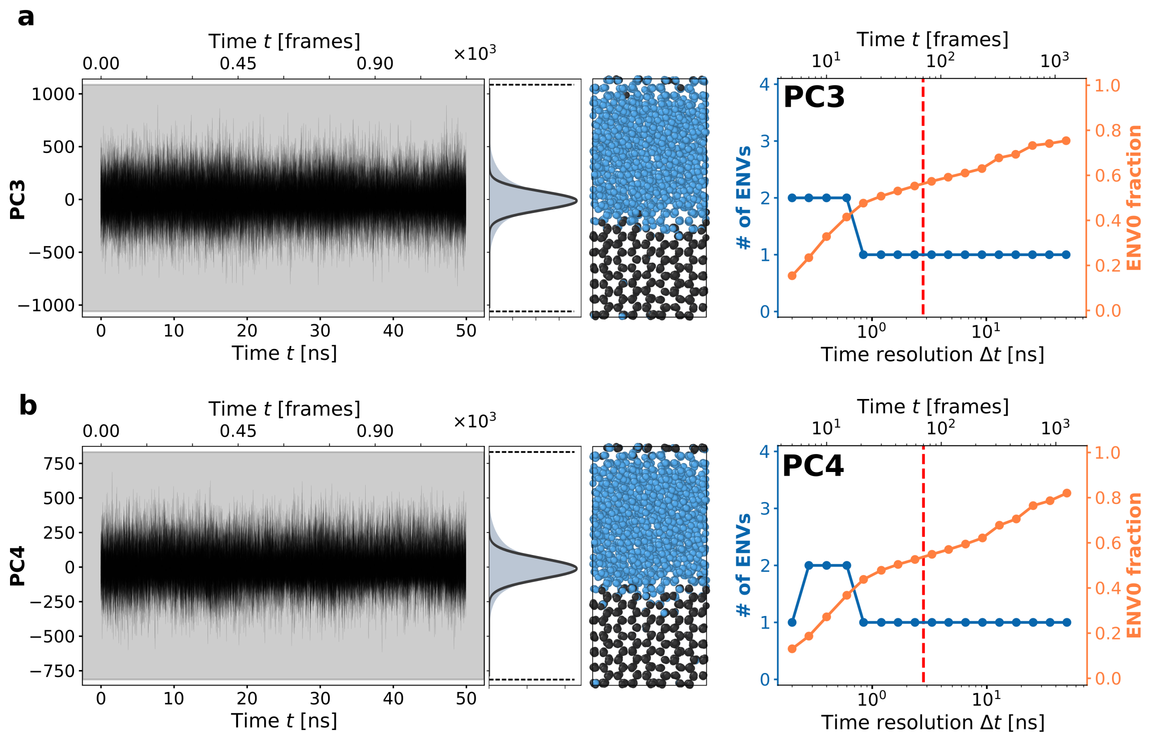


Figure S3: **Onion Clustering on PC3 and PC4 time-series data.** a) From left to right: PC3 time-series data of all molecules; kernel density estimate (KDE) of the PC3 time-series data; screenshot of the MD simulation, with molecules colored according to Onion Clustering micro-clusters; Onion output plot. b) Same as in a) but for PC4.

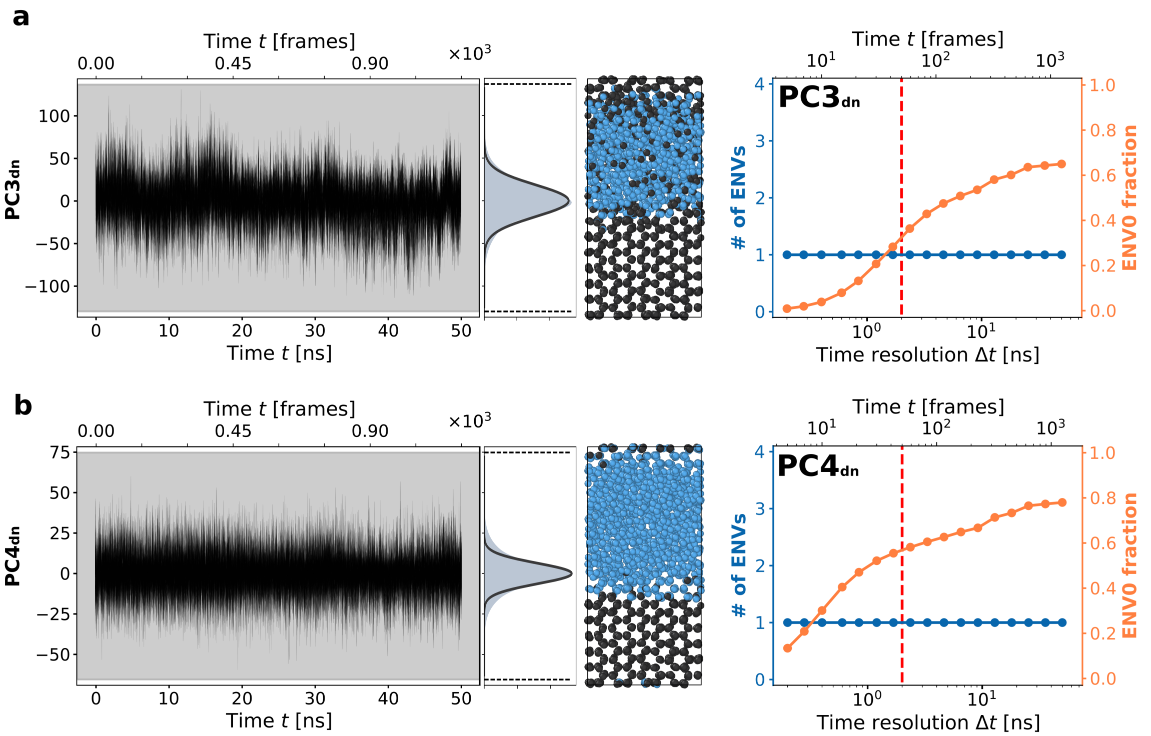


Figure S4: **Onion Clustering on denoised PC3 and PC4 time-series data.** The figure follows the same structure of Figure S3.

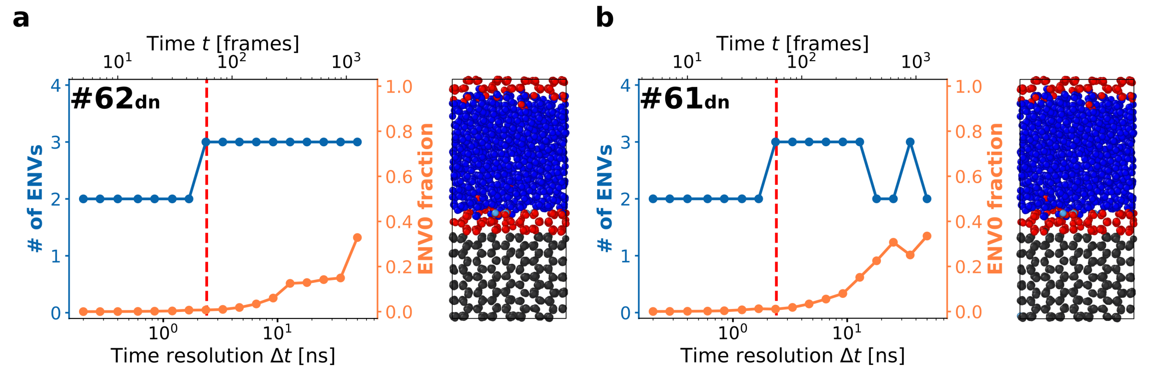


Figure S5: **Onion Clustering on denoised #62 and #61 time-series data.** a) Onion plot of $\#62_{dn}$ and snapshots of the MD trajectory colored according to Onion micro-clusters. b) Same as a) but for $\#61_{dn}$.

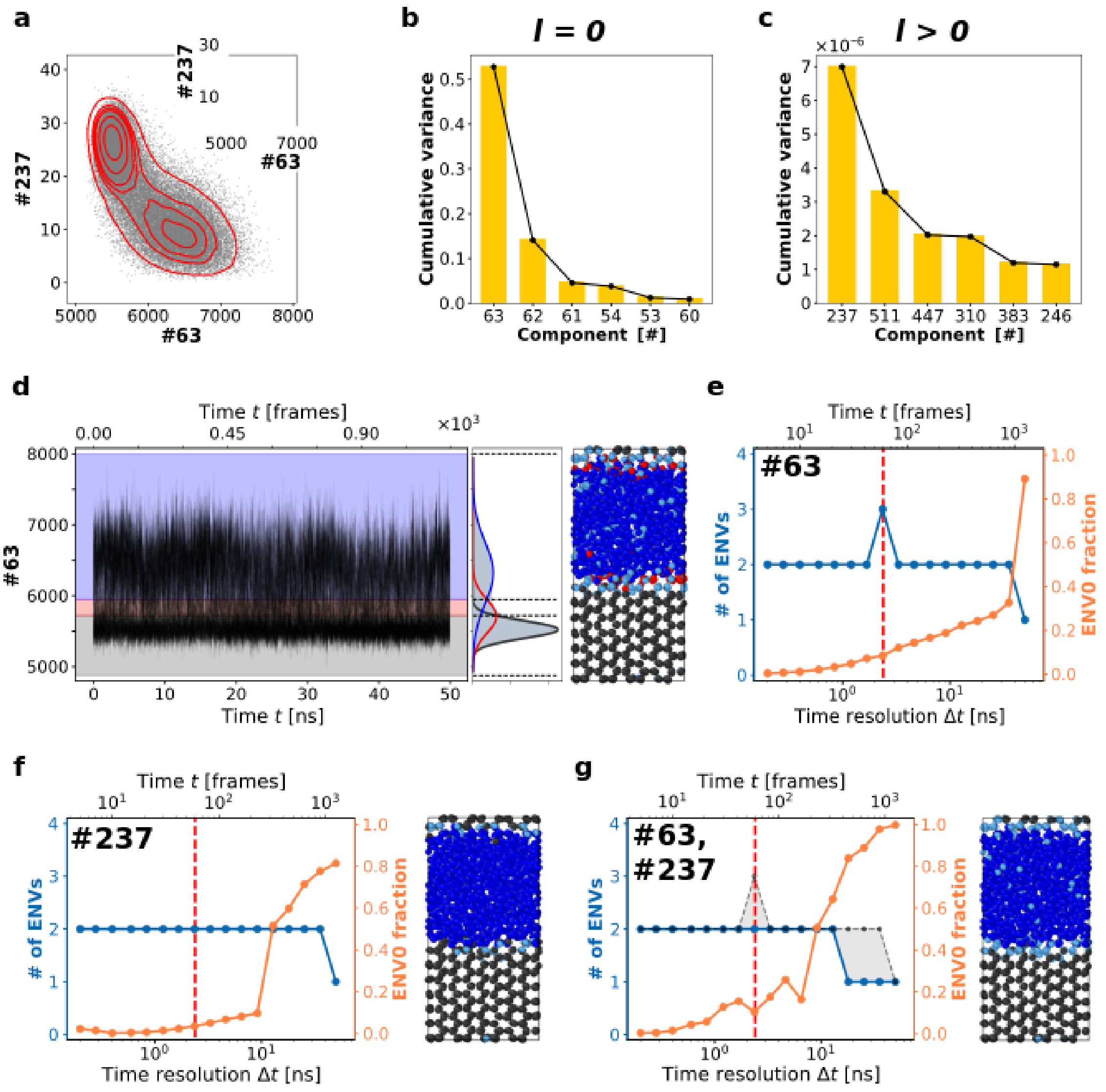


Figure S6: **Onion Clustering on raw #63 and #237 time-series data.** a) Denoised dataset projection onto components #63 and #237, red contour lines help visualize the data density; in the inset, static clustering distinguishes 2 environments. b) Variance of the six most significant spherical ($l = 0$) SOAP components. c) Variance of the six most significant non-spherical ($l > 0$) SOAP components. d), e) Onion Clustering results of component #63. f) Onion Clustering results of component #237. g) Bi-dimensional Onion Clustering results of components #63, #237.

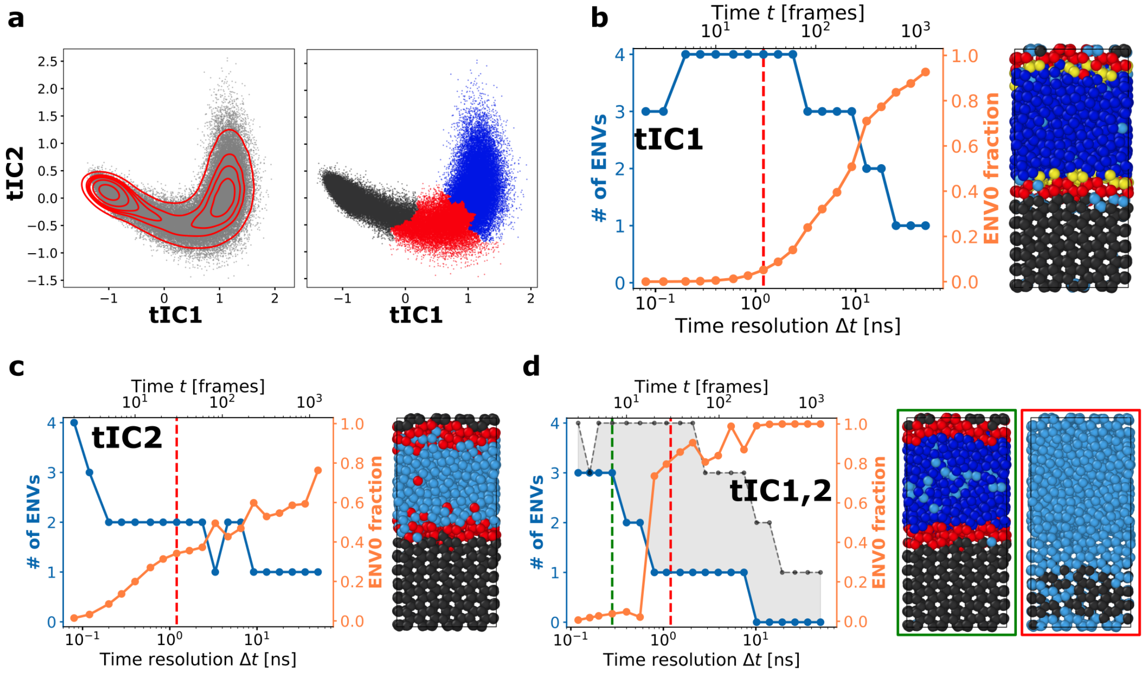


Figure S7: **Onion Clustering on raw Time-lagged Independent Component Analysis (tICA) [1, 2] components tIC1 and tIC2.** a) right: Dataset projection onto components tIC1 and tIC2, red contour lines help visualize the data density; left: static clustering distinguishes 3 environments. b) Onion Clustering results on tIC1. d) Onion Clustering results on tIC2. d) Bi-dimensional Onion Clustering results of tIC1, tIC2. tIC1 is able to clearly discriminate ice from water and two layers of interface, while tic2 distinguishes ice bulk and the interface, while water is assigned to ENV0. The bi-dimensional Onion Clustering clearly demonstrate the effect of frustrated information.

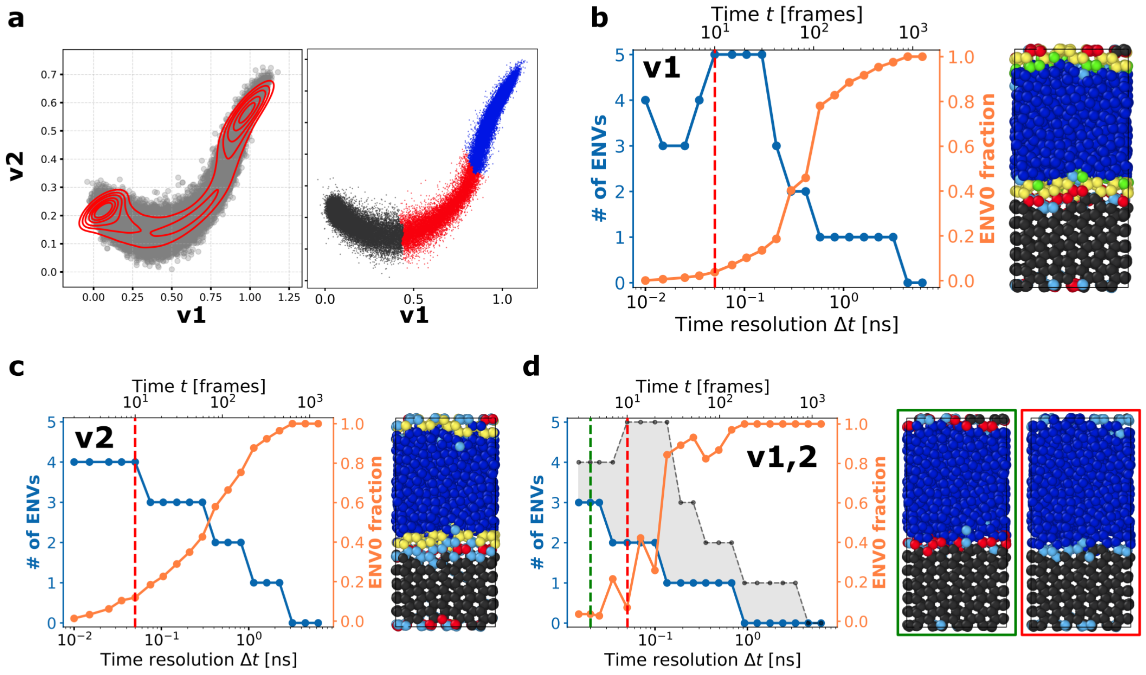


Figure S8: **Comparative VAMPnets [3] results.** a) Left: Projection of the SOAP dataset on the components VMAPnets information-richest v1 and v2 components space: red contour isolines help visualizing the data density. Right: hierarchical clustering conducted on the entire SOAP dataset (ergodic analysis, no time-correlation between the data) allows distinguishing 3 environments. b) Onion Clustering results on v1 time-series data: this allows resolving up to 5 distinct microclusters. c) Onion Clustering results on v2 time-series data: up to 4 distinct microclusters can be resolved. Comparison of b-c) *vs.* a) shows how, also in this case, there is more information in the individual components v1 and v2 over time than in v1+v2 taken together, but without considering the information contained in the time-correlation between their data. In particular, v1 alone contains information allowing to discriminate the ice and water phases, and three interface layers (the center of the interface, plus two interlayers along which the molecules exchange between the bulk of ice or of water with the inner part of the interface). v2 contains information allowing to distinguish ice and water bulk phases, and two interface environments. d) Bi-dimensional Onion Clustering results of the bivariate v1, v2 time-series data. Also in this case, such bi-dimensional Onion Clustering analysis is affected by information frustration effects: when the v1 and v2 time-series are clustered together, the information that these contain "disturb" each other, leading to information loss. At best, at high resolution such analysis allows resolving three separated clusters - as the pattern recognition analysis of panel a), suggesting that due to such information frustration effects, the useful information contained in the time-correlations of the data contained along v1 and v2 (taken separately) are lost.

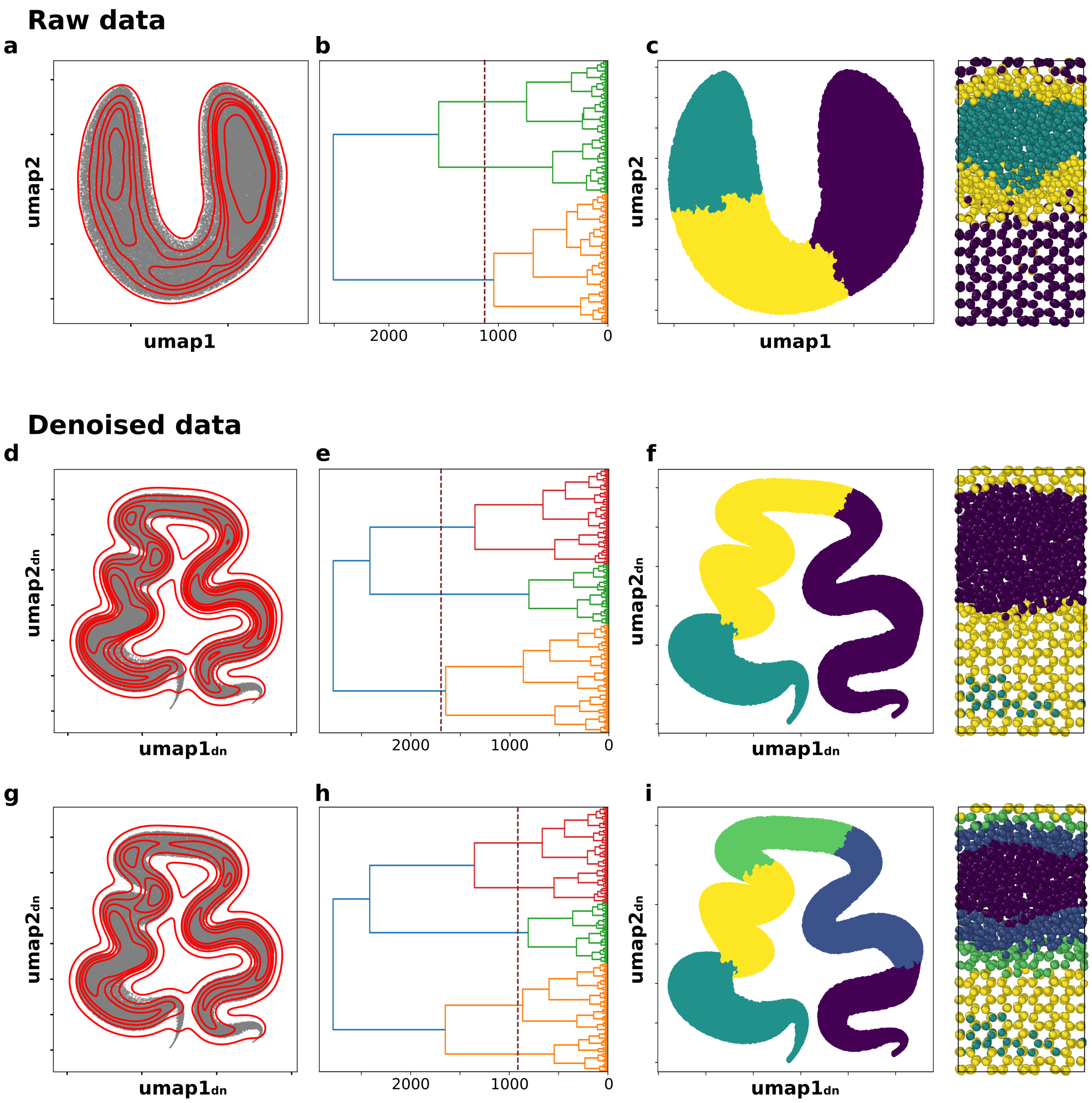


Figure S9: **UMAP results on raw and denoised SOAP dataset**. a) Projection of the raw SOAP dataset on the first two UMAP components. Red contour lines help visualize the data density. b) Dendrogram obtained by applying Hierarchical clustering on UMAP1, UMAP2. c) Right: dataset colored according to the clustering results from the Hierarchical clustering. Left: Snapshots of the MD trajectory, colored according to the micro-clusters detected. d)- i) Results of the Hierarchical clustering applied on the UMAP dataset obtained for denoised SOAP spectra. d)-f) Propose the results when cutting the dendrogram at three clusters. g)-i) Propose the results when cutting the dendrogram at five clusters.

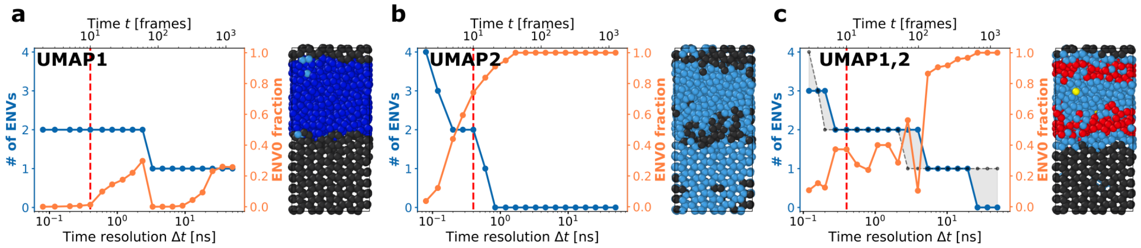


Figure S10: **Onion Clustering on denoised UMAP1 and UMAP2**. Onion Clustering results of a) UMAP1$_{dn}$, b) UMAP2$_{dn}$, and c) combination of UMAP1$_{dn}$ and UMAP2$_{dn}$. Differently from other cases studied herein, the combination of the two features enhance the analysis as a consequence of the shape of the dataset, which is clearly be dimensional, and then requires both the variables to detect all the environments.

# References

(1) Molgedey, L.; Schuster, H. G. Separation of a mixture of independent signals using time delayed correlations. *Physical Review Letters* **1994**, *72*, 3634–3637.

(2) Pérez-Hernández, G.; Paul, F.; Giorgino, T.; De Fabritiis, G.; Noé, F. Identification of slow molecular order parameters for Markov model construction. *The Journal of Chemical Physics* **2013**, *139*, 015102.

(3) Mardt, A.; Pasquali, L.; Wu, H.; Noé, F. VAMPnets for deep learning of molecular kinetics. *Nature Communications* **2018**, *9*, DOI: 10.1038/s41467-017-02388-1.
